# Treatments for Primary Delusional Infestation: Systematic Review

**DOI:** 10.2196/34323

**Published:** 2022-03-30

**Authors:** Justin Di Lu, Ryan D Gotesman, Shawn Varghese, Patrick Fleming, Charles W Lynde

**Affiliations:** 1 Michael G DeGroote School of Medicine Hamilton, ON Canada; 2 Faculty of Medicine University of Ottawa Ottawa, ON Canada; 3 Division of Dermatology University of Toronto Toronto, ON Canada; 4 Lynde Dermatology Markham, ON Canada

**Keywords:** delusional infestation, Morgellons disease, treatment, delusional parasitosis, atypical, typical, antipsychotic, SSRI, delusion, rare disorder, systematic review, pharmacology, pharmacological, psychiatric, dermatology, dermatologist, drug

## Abstract

**Background:**

Delusional infestation, also known as Ekbom syndrome, is a rare delusional disorder characterized by the fixed belief that one is infested with parasites, worms, insects, or other organisms. Although delusional infestation is a psychiatric condition, patients often consult dermatologists with skin findings, and it is currently unclear what treatments are recommended for this disorder.

**Objective:**

We aimed to systematically review and describe the treatment and management of patients presenting with primary delusional infestation.

**Methods:**

A systematic search was conducted using Ovid on MEDLINE, Embase, PsycINFO, and the Cochrane Register of Clinical Trials. Relevant data, including treatment, dosage, response, adherence, and side effects, were extracted and analyzed.

**Results:**

A total of 15 case series were included, comprising 280 patients (mean age 53.3 years, 65.4% female) with delusional infestation. Overall, aripiprazole had the highest complete remission rate at 79% (11/14), although this was limited to 14 patients. Among drug classes, selective serotonin reuptake inhibitors were the most effective with a 79% (11/14) complete remission rate and 43% (9/21) partial remission rate in patients with comorbid depression, anxiety, or trichotillomania. First-generation antipsychotics and second-generation antipsychotics had similar complete remission rates (56/103, 54.4% vs 56/117, 47.9%, respectively) and partial remission rates (36/103, 35% vs 41/117, 35%, respectively).

**Conclusions:**

Due to the rarity of delusional infestation, we only found 15 case series. However, we found that first-generation antipsychotics appear to be similar in effectiveness to second-generation antipsychotics for the treatment of primary delusional infestation. Larger studies and randomized controlled trials are needed to evaluate the efficacy of pharmacological therapy for delusional infestation.

**Trial Registration:**

PROSPERO CRD42020198161; https://www.crd.york.ac.uk/prospero/display_record.php?RecordID=198161

## Introduction

Delusional infestation, also known as delusional parasitosis, is a rare delusional disorder characterized by the fixed belief that one’s skin is infested by parasites, worms, insects, or other organisms [1]. The prevalence of delusional infestation is estimated to be 27.3 per 100,000, and it is more frequent in individuals over the age of 50 years and in socially isolated women [2,3]. Despite the lack of microbiological evidence, patients are convinced they are infected and often present with cutaneous sensations, such as itching, crawling, and formication. These delusions may lead patients to injure themselves through cuts and chemical burns or destroy their furniture in an attempt to eliminate the perceived infestation [4]. The “specimen sign” is a classic feature of the illness present in about half of all patients, in which patients present fragments of skin, particles, threads, or insects to their healthcare provider as evidence of skin infestation [5].

Delusional infestation can be classified as either a primary or secondary variant. Primary delusional infestation is an isolated psychiatric disorder diagnosed after the exclusion of other causes, such as infection or an underlying medical or psychiatric condition. In secondary delusional infestation, the delusions are attributed to other conditions, including substance use, medications, other psychiatric conditions, and infections. Primary delusional infestation comprises approximately 56% of cases [6].

The etiology of primary delusional infestation is unclear, though disruptions in dopamine pathways are suspected to play a role. Antipsychotics improve delusional infestation symptoms, likely due to inhibition of dopamine transmission. Dopamine plays a role in probabilistic reasoning, and its disruption may cause patients to incorrectly attribute a rash or itch to skin infestation [1,7]. Another hypothesis suggests that dysfunction of striatal dopamine transporters leads to more postsynaptic dopamine, increasing the risk of developing delusional infestation [8]. Conditions associated with reduced dopamine transporter function, such as schizophrenia, depression, and alcoholism, have been associated with delusional infestation. Moreover, medications that inhibit dopamine reuptake, such as cocaine and amphetamines, often induce delusional infestation symptoms, such as formication [8]. There is also evidence that dysfunction in the fronto-striato-thalamic network mediates symptoms of delusional infestation [9].

The clinical management of delusional infestation is challenging, and dermatologists are often consulted due to patients conceptualizing the disease as somatic. Patients frequently refuse psychiatric therapy or referral and often present proof of infestation, which is commonly referred to as a “specimen sign” or “matchbox sign” and can include skin particles or hair. On average, dermatologists will manage 2 to 3 patients with delusional infestation every 5 years [10]. Common treatments reported in the literature include first-generation antipsychotics (FGAs) (eg, pimozide, fluphenazine, and haloperidol) and second-generation antipsychotics (SGAs) (eg, risperidone and olanzapine). A 2007 systematic review of papers on delusional infestation found FGAs and SGAs were effective in the majority of patients with primary delusional infestation, but remission rates did not differ between these 2 groups of antipsychotics [11]. A more recent systematic review reported similar results; there was no strong evidence to suggest any single antipsychotic agent over another [12]. Both of these reviews restricted their search strategy to antipsychotics; however, other pharmacological agents may also prove effective in treating delusional infestation. As such, we conducted a systematic review to identify pharmacological treatments used for primary delusional infestation to better understand their effectiveness and establish recommendations for the management of primary delusional infestation.

## Methods

**Figure 1:**
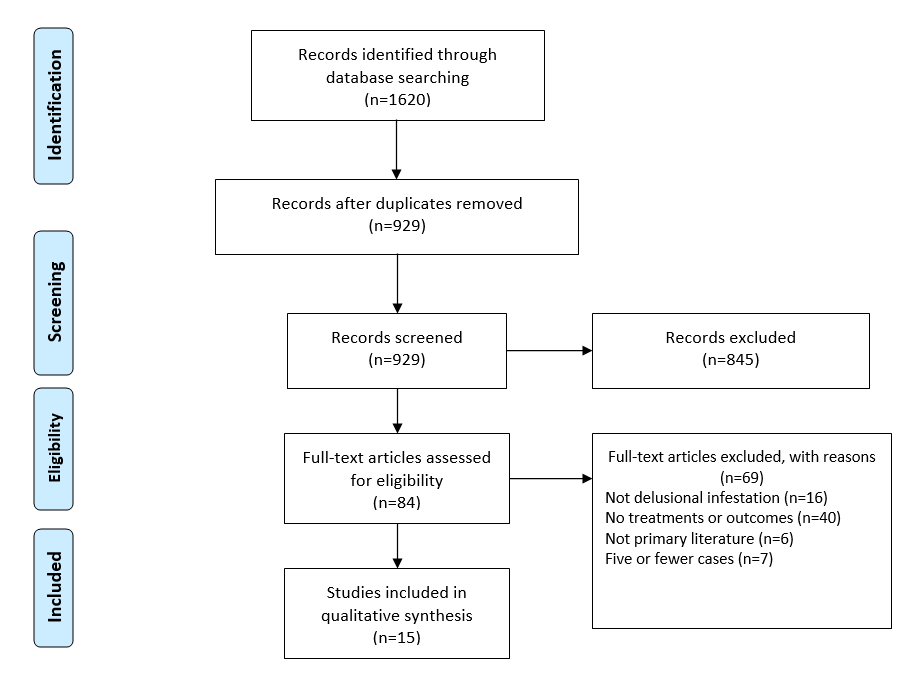
PRISMA (Preferred Reporting Items for Systematic Reviews and Meta-Analyses) flow diagram for identifying cases of delusional infestation.

The protocol was registered on PROSPERO (CRD42020198161). The PRISMA (Preferred Reporting Items for Systematic Reviews and Meta-Analyses) guidelines were utilized in this systematic review (Figure 1).

### Search Strategy and Data Extraction

A systematic search was conducted using Ovid on MEDLINE, Embase, PsycINFO, and the Cochrane Register of Clinical Trials from June 2020. The full search strategy is detailed in Textbox 1. Eligibility for inclusion of articles was established a priori. Articles were included if they (1) were written in English and (2) were original articles that evaluated pharmacological treatments for delusional infestation. Articles were excluded if they (1) were nonoriginal articles (eg, conference abstracts or reviews), (2) evaluated fewer than 5 patients (eg, case reports), or (3) did not evaluate pharmacological treatments. All keywords were searched and mapped onto subject headings where appropriate. References of included studies were screened for inclusion.

Screening of titles and abstracts was independently conducted by 2 reviewers (JDL and RDG) and was followed by a full text review. Discrepancies were resolved through consensus or by consulting the corresponding author (CL).

Variables related to general study data, including article title, journal, authors, year of publication, study design, and the number of cases were collected by 2 independent reviewers (JDL and RDG). Variables related to clinical information were also collected, including mean age, proportion of female patients, reported pathogens, psychiatric family history, co-occurring dermatological conditions, treatments (including placebo), dosage, treatment duration, treatment outcomes (including full remission, partial remission, no response, and nonadherence), and side effect profiles.

**Search strategy for studies on delusional infestation.**

1. Delusional Parasitosis.mp. or Delusional Parasitosis/

2. Morgellons Disease.mp. or Morgellons Disease/

3. Delusional infestation.mp.

4. Dermatozoic delusion.mp.

5. Delusory parasitosis.mp.

6. Delusions of parasitosis.mp.

7. Psychogenic parasitosis.mp.

8. Ekbom syndrome.mp.

9. Dermatophobia.mp.

10. Parasitophobia.mp.

11. Cocaine bugs.mp.

12. Chronic tactile hallucinosis.mp.

13. Acarophobia

14. Monosymptomatic hypochondriacal psychosis

15. 1 or 2 or 3 or 4 or 5 or 6 or 7 or 8 or 9 or 10 or 11 or 12 or 13 or 14

### Risk of Bias

The quality of the included studies was appraised independently by 2 reviewers (JDL and RDG) with a formal risk of bias assessment. The National Institutes of Health (NIH) quality assessment tool for case series was used to evaluate the risk of bias in the included studies. Disagreements were resolved by consensus and discussion with a third reviewer (SV) as necessary. Eligible studies were excluded if they contained a high risk of bias.

### Data Analysis

Data were reported as means, frequency, or proportions as needed. Study characteristics and outcome data were recorded, including the number of treatments and treatment efficacy (based on the categories of complete remission, partial remission, no response, and nonadherence). We assigned individual outcomes to 3 main categories: no response, partial remission (ie, some response), and full remission. Efficacy of treatment was synthesized by dividing the total number of patients with a certain response by the total number receiving treatment [11].

## Results

### Included Studies

A total of 1620 studies were identified by searching the databases and additional references (Textbox 1); 691 articles were duplicates, leaving 929 studies for title and abstract screening. After screening, 84 articles underwent full-text review. Next, 69 articles were excluded due to not involving delusional infestation (n=16), not reporting treatments or outcomes (n=40), not being primary literature (n=6), and having fewer than 5 cases (n=7). A total of 15 articles met the inclusion criteria and were included in the systematic review (Table 1) [13-27]. Most of the available studies had low methodological quality due to small sample sizes or having an uncontrolled or retrospective design, so a meta-analysis was not conducted.

**Table 1:** Demographics and characteristics of included studies of primary delusional infestation.

	Author, year	Size	Mean age (years)	Sex (% female, n/N)	Reported pathogens	Psychiatric history	Co-occurring dermatological conditions	Comorbid conditions	Treatments	Side effects of treatments
1	Frithz, 1979 [13]	15	58.2	93% (14/15)	Parasites	None	—^a^	—	Fluphenazine, flupentixol	Extrapyramidal symptoms in half of patients, relieved with orphenadrine hydrochloride.
2	Sheppard et al, 1986 [14]	8	55.4	38% (3/8)	Lice, fleas, insects	Depression (n=1), social isolation (n=1)	Pruritus vulvae (n=1)	—	Pimozide	—
3	Srinivasa et al, 1994 [15]	19	40.4	63% (12/19)	—	—	—	—	Trifluoperazine,chlorpromazine, haloperidol	—
4	Räsänen et al, 1997 [16]	6	74.5	100% (6/6)	Fleas, insects, worms, lice	Insomnia (n=2), depression (n=2), anxiety (n=2), social isolation (n=1)	—	None	Perphenazine, haloperidol, melperone, citalopram, zuclopenthixol, sertraline	None
5	Zanol et al, 1998 [17]	20	40	55% (11/20)	Parasites	—	Ichthyosis vulgaris (n=1), scabies, body lice, crab lice (n=6)	T2DM^b^ (n=2), renal failure (n=1), chronic hepatitis C infection (n=1)	Pimozide, alprazolam, doxepin, ativan, imipramine, haloperidol	—
6	Bhatia et al, 2000 [18]	52	54.5	64% (33/52)	Insects (n=23, 44%)	Adjustment disorder (n=1), trichotillomania (n=3), dementia (n=5), depression (n=4)	—	T2DM (n=2), leprosy (n=5)	Imozide, fluoxetine, amitriptyline	—
7	Zomer et al, 2002 [19]	18	56.9	61% (11/18)	Pests or fleas	—	—	—	Pimozide	—
8	Nicolato et al, 2006 [20]	10	72.4	70% (7/10)	Parasites	Depression (n=2), dementia, (n=2), schizophrenia (n=1)	—	T2DM (n=2), hypertension (n=2), thyroid disease (n=3), COPD^c^ (n=1), heart failure (n=2)	Risperidone, haloperidol, olanzapine, pimozide, quetiapine, rivastigmine	—
9	Ahmad and Ramsay, 2009 [21]	10	41.9	60% (6/10)	Insects, bugs, viruses, mites, black things	Depression (n=4)	History of scabies (n=7)	—	Pimozide, sulpiride	—
10	KenchaiaH et al, 2009 [22]	20	49.8	—	—	—	—	—	Haloperidol, risperidone olanzapine, fluoxetine, sertraline, imipramine	—
11	Coşar et al, 2012 [23]	10	61.7	80% (8/10)	—	—	—	Hypertension (n=4), COPD (n=2)	Pimozide, olanzapine, risperidone	—
12	Bhatia et al, 2013 [24]	50	—	66% (33/50)	Insects (n=28, 56%)	Depression (n=5), dementia (n=2), trichotillomania (n=4)	Alopecia (n=3)	T2DM (n=2), leprosy (n=3)	Risperidone, olanzapine, amisulpride, quetiapine, aripiprazole, paliperidone, iloperidone, fluoxetine	—
13	MohandaS et al, 2017 [25]	28	54.6	71% (20/28)	Fibers, fungi, dust, bugs, grains, black dots, parasites	Depression (n=12), anxiety (n=7)	—	—	Risperidone, olanzapine	Olanzapine-induced weight gain (n=2)
14	Çınar et al, 2019 [26]	8	57.5	38% (3/8)	—	—	—	Hypertension (n=4)	Aripiprazole	None
15	Jerrom et al, 2019 [27]	6	—	50% (3/6)	Black bits, fibers	Anxiety and depression (n=2), PTSD (n=1)	—	None	Risperidone, aripiprazole	None

^a^Em dashes indicate “not reported.”

^b^T2DM: type 2 diabetes.

^c^COPD: chronic obstructive pulmonary disease.

### Risk of Bias

The risk of bias assessment is presented in Table 2. Overall, the studies had a low risk of bias based on the NIH quality assessment tool. Out of the 15 included studies, 13 were rated “good” overall and 2 were rated “fair” based on the 9 criteria.

**Table 2:** Risk of bias assessment using the National Institutes of Health quality assessment tool for case series studies.

Study	Author, year	Criteria									
		1. Was the study question or objective clearly stated?	2. Was the study population clearly and fully described, including a case definition?	3. Were the cases consecutive?	4. Were the subjects comparable?	5. Was the intervention clearly described?	6. Were the outcome measures clearly defined, valid, reliable, and implemented consistently across all study participants?	7. Was the length of follow-up adequate?	8. Were the statistical methods well-described?	9. Were the results well-described?	Overall rating (good, fair, poor)
1	Frithz, 1979 [13]	Yes	Yes	Yes	Yes	Yes	Yes	Yes	Yes	Yes	Good
2	Sheppard et al, 1986 [14]	Yes	Yes	Yes	Yes	Yes	Yes	Yes	Yes	Yes	Good
3	Srinivasa N et al, 1994 [15]	Yes	Yes	No	Yes	Yes	Yes	Yes	Yes	Yes	Good
4	Räsänen et al, 1997 [16]	Yes	Yes	No	Yes	Yes	Yes	No	Yes	Yes	Fair
5	Zanol et al, 1998 [17]	Yes	Yes	—^a^	Yes	Yes	Yes	Yes	—	Yes	Fair
6	Bhatia et al, 2000 [18]	Yes	Yes	Yes	Yes	Yes	Yes	—	Yes	Yes	Good
7	Zomer et al, 2002 [19]	Yes	Yes	Yes	Yes	Yes	Yes	—	Yes	Yes	Good
8	Nicolato et al, 2006 [20]	Yes	Yes	Yes	Yes	Yes	Yes	Yes	Yes	Yes	Good
9	Ahmad and Ramsay, 2009 [21]	Yes	Yes	Yes	Yes	Yes	Yes	Yes	Yes	Yes	Good
10	Kenchaia H et al, 2009 [22]	Yes	Yes	Yes	Yes	Yes	Yes	Yes	Yes	Yes	Good
11	Coşar et al, 2012 [23]	Yes	Yes	Yes	Yes	Yes	Yes	Yes	Yes	Yes	Good
12	Bhatia et al, 2013 [24]	Yes	Yes	Yes	Yes	Yes	Yes	Yes	Yes	Yes	Good
13	Mohandas et al, 2017 [25]	Yes	Yes	Yes	Yes	Yes	Yes	—	Yes	Yes	Good
14	Çınar et al, 2019 [26]	Yes	Yes	Yes	Yes	Yes	Yes	Yes	Yes	Yes	Good
15	Jerrom et al, 2019 [27]	Yes	Yes	Yes	Yes	Yes	Yes	—	Yes	Yes	Good

^a^Em dashes indicate “not applicable.”

### Study Characteristics

The 15 articles identified were all case series and included an overall total of 280 patients with primary delusional infestation. The mean age was 53.3 years and the patients were preponderantly female (170/260, 65.4%) (Table 1). The most commonly reported pathogens were insects, parasites, black specks, lice, and fibers. Across all 15 articles, psychiatric history was unreported in almost half of the studies (7/15, 47%); however, in articles that did report psychiatric history, anxiety had the highest reported rate (11/40, 27%), followed by depression (32/162, 19.8%), insomnia (2/6, 33%), posttraumatic stress disorder (1/6, 16%), social isolation (2/14, 14%), schizophrenia (1/10, 10%), dementia (9/112, 8.0%), and trichotillomania (7/102, 6.9%). A history of scabies or lice was noted in 43% (13/30) of patients. Family history and comorbidities were generally not reported. Pharmacological treatments included 2 antidepressants (fluoxetine and citalopram), 8 FGAs (pimozide, haloperidol, fluphenazine depot, trifluoperazine, flupentixol depot, chlorpromazine, perphenazine, and zuclopenthixol), and 9 SGAs (risperidone, olanzapine, aripiprazole, quetiapine, amisulpride, paliperidone, iloperidone, melperone, and sulpiride). Side effects of the treatments were generally not reported, with the exception of fluphenazine- and flupentixol-induced extrapyramidal symptoms in 7 patients, which was relieved with orphenadrine hydrochloride, reported in the paper by Frithz [13], and olanzapine-induced weight gain in 2 patients in the report by Mohandas et al [25].

### Efficacy of FGAs

A summary of the pharmacological treatments for primary delusional infestation is outlined in Table 3. The 3 main classes of drugs were selective serotonin reuptake inhibitors (SSRIs) (n=2), FGAs (n=8), and SGAs (n=9). Across the 15 studies, 8 kinds of FGA were used by a total of 117 patients. The treatment duration ranged from 0.75 to 14 months and 47.9% (56/117) of patients achieved complete remission, 35% (41/117) achieved partial remission, and 17.1% (20/117) had no response or were nonadherent. Pimozide, haloperidol, and fluphenazine depot were the most common FGAs prescribed. A total of 80 patients received pimozide, with a dose ranging from 2 to 8 mg/d; 44% (35/80) achieved complete remission, while 34% (27/80) achieved partial remission and 23% (18/80) had no response. Haloperidol (dosage: 1 to 10 mg) led to 60% (6/10) complete remission and 40% (4/10) partial remission and fluphenazine depot (dosage: 7.5 to 25 mg/d) resulted in 70% (7/10) complete remission and 30% (3/10) partial remission, but both drugs were limited to a small sample size of 10 patients. The remaining FGAs were each used to treat fewer than 10 patients and included trifluoperazine, flupentixol depot, chlorpromazine, perphenazine, and zuclopenthixol (Table 3).

**Table 3:** Summary of pharmacological treatments for primary delusional infestation.

Drug				Dose (mg/d)		Total number of patients, N		Duration, months		Outcomes						
										Complete remission, n (%)		Partial remission, n (%)		No response, n (%)		Nonadherence, n (%)
**First-generation antipsychotics (n=8)**																
	Total		—^a^		117		0.75-14		56 (47.9)		41 (35.0)		20 (17.1)		0 (0)	
	Pimozide		2-8		80		3-14		35 (44)		27 (34)		18 (23)		0 (0)	
	Haloperidol		1-10		10		0.75-14		6 (60)		4 (40)		0 (0)		0 (0)	
	Fluphenazine depot		7.5-25		10		3-12		7 (70)		3 (30)		0 (0)		0 (0)	
	Trifluoperazine		10, 15		6		0.75-2		3 (50)		2 (33)		1 (17)		0 (0)	
	Flupentixol depot		2-20		5		3-12		4 (80)		0 (0)		1 (20)		0 (0)	
	Chlorpromazine		150, 300		3		0.75-2		0 (0)		3 (100)		0 (0)		0 (0)	
	Perphenazine		4,12		2		—		1 (50)		1 (50)		0 (0)		0 (0)	
	Zuclopenthixol		6		1		—		0 (0)		1 (100)		0 (0)		0 (0)	
**Second-generation antipsychotics (n=9)**																
	Total		—		103		3-24		56 (54.4)		36 (35.0)		8 (7.8)		3 (2.9)	
	Risperidone		0.5-4		44		3-24		19 (43)		18 (41)		5 (11)		2 (5)	
	Olanzapine		2.5-10		22		3-24		12 (55)		9 (41)		1 (5)		0 (0)	
	Aripiprazole		10-15		14		3-24		11 (79)		2 (14)		0 (0)		1 (7)	
	Quetiapine		100, 400		7		6-24		4 (57)		3 (43)		0 (0)		0 (0)	
	Amisulpride		—		7		6-24		3 (43)		3 (43)		1 (14)		0 (0)	
	Paliperidone		—		5		6-24		4 (80)		1 (20)		0 (0)		0 (0)	
	Iloperidone		—		2		6-24		2 (100)		0 (0)		0 (0)		0 (0)	
	Melperone		50		1		—		1 (100)		0 (0)		0 (0)		0 (0)	
	Sulpiride		—		1		—		0 (0)		0 (0)		1 (100)		0 (0)	
**Selective serotonin reuptake inhibitors (n=2)**																
		Total		—		21		6-24		12 (57)		9 (43)		0 (0)		0 (0)
		Fluoxetine		20		11		6-24		9 (82)		2 (18)		0 (0)		0 (0)
		Citalopram		—		10		—		3 (30)		7 (70)		0 (0)		0 (0)

^a^Em dashes indicate “not reported”.

### Efficacy of SGAs

Overall, 9 kinds of SGA were used by 103 patients. The treatment duration ranged from 3 to 24 months, and 54.4% (56/103) of patients achieved complete remission, 35% (36/103) achieved partial remission, and 10.7% (11/103) had no response or were nonadherent (Table 3). The most common SGAs prescribed were risperidone, olanzapine, and aripiprazole. Of 43 patients on risperidone (dosage: 0.5 to 4 mg/d), 43% (19/44) achieved complete remission, 41% (18/44) achieved partial remission, and 16% (7/44) had no response. Of 22 patients on olanzapine (dosage: 2.5 to 10 mg/d), 55% (12/22) achieved complete remission, 41% (9/22) achieved partial remission, and 5% (1/22) had no response. Of 14 patients on aripiprazole (dosage: 10 to 15 mg/d), 79% (11/14) achieved complete remission, 14% (2/14) achieved partial remission, and 7% (1/14) were nonadherent. The remaining 6 SGAs were each used to treat fewer than 10 patients and included quetiapine, amisulpride, paliperidone, iloperidone, melperone, and sulpiride (Table 3).

### Efficacy of SSRIs

Overall, 2 kinds of SSRI were used. Fluoxetine was used by 11 patients and citalopram was used by 10 patients. These SSRIs were used to treat comorbid depression, anxiety, and trichotillomania. Trichotillomania might also have been a secondary delusional infestation, although this was not specified in these studies. Treatments were effective, with an overall 57% (12/21) complete remission rate and 43% (9/21) partial remission rate (Table 3). Fluoxetine appeared to be more efficacious, with 82% (9/21) complete remission and 18% (2/21) partial remission, compared to citalopram with 30% (3/10) complete remission and 70% (7/10) partial remission.

## Discussion

### Principal Findings

We conducted a systematic review of studies on pharmacological treatments for primary delusional infestation. Psychiatric history was unreported by almost half the studies, but of the remaining studies, the most commonly reported psychiatric disorders were anxiety (11/40, 28%) and depression (32/162, 20%). The efficacy of the drug classes used in the studies varied; 57.1% (12/21) of patients who received SSRIs had complete remission and 42.9% (9/21) had partial remission, 54.4% (56/103) of patients who received SGAs had complete remission, 35% (36/103) had partial remission, and in 10.7% (11/103) of patients, the treatment was not effective, due to either nonresponse or nonadherence. Among patients (n=117) who received FGAs, 47.9% (56/117) had complete remission, 35% (41/117) had partial remission, and the treatment was not effective in 17.1% (20/117) of patients.

Although antipsychotics are the mainstay in the treatment of primary delusional infestation, no antipsychotics are approved for this use and there is no strong evidence suggesting that the use of any specific antipsychotic is more effective than any other [10,28,29]. We compared FGAs and SGAs and found that patients using SGAs had higher rates of complete remission and lower rates of noneffectiveness than patients using FGAs. A 2020 systematic review by McPhie and Kirchhof [12] similarly concluded there was no strong evidence to recommend any one antipsychotic over another, due to a low quality of evidence and study variability.

While the efficacy of both FGAs and SGAs is comparable, these agents vary in their side effect profiles. FGAs are known to produce extrapyramidal side effects, including parkinsonism, acute dystonia, akathisia, and tardive dyskinesia. While some of these side effects may be controlled with additional pharmacotherapy, extrapyramidal side effects can decrease quality of life, decrease compliance, lead to polypharmacy, and may even be permanent (eg, tardive dyskinesia) [30]. By contrast, SGAs generally have a lower incidence of extrapyramidal side effects, but their efficacy and side effect profiles vary widely based on the specific agent [31-33]. Given the higher rates of complete remission and lower rates of noneffectiveness that we found for SGAs compared to FGAs in this study, as well as the more variable side effect profiles of SGAs, SGAs may be more beneficial in the treatment of primary delusional infestation. However, all the studies included were case series, and in the absence of higher levels of evidence, such as that provided by randomized controlled trials, we can only draw conclusions and make recommendations with caution. Further studies should be conducted.

Risperidone is the most widely studied SGA, followed by olanzapine [12]. Although olanzapine had a higher complete remission rate and lower noneffectiveness rate compared to risperidone in our study, olanzapine is known to have a higher incidence of metabolic side effects, such as weight gain, relative to other SGAs [34]. Interestingly, we found that aripiprazole had the highest complete remission rate (11/14, 79%) compared to both risperidone (19/44, 43%) and olanzapine (12/22, 55%). Furthermore, aripiprazole is known to have a lower rate of metabolic side effects than other SGAs [35] and has the additional advantage of acting as a partial dopamine agonist [36], making it a useful adjunct in the treatment of depression, which is a common comorbidity in patients with delusional infestation. While these results are promising for the use of aripiprazole in delusional infestation, further studies are required before its use can be widely recommended.

Interestingly, the majority of patients treated with SSRIs had complete remission of delusional infestation, although this was limited to a sample size of 21 patients with comorbid depression, anxiety, or trichotillomania in 3 studies [18,24,25]. These patients were managed with fluoxetine or citalopram. This suggests that clinicians should obtain a full psychiatric history of patients with delusional infestation to identify underlying mood and anxiety disorders that might respond to SSRIs, thereby improving the management of this challenging illness.

### Limitations

Due to the rarity of delusional infestation, there is a lack of clinical trials and cohort studies, and our analysis included only case series studies, all of which used subjective measures of treatment efficacy. In addition, we only assessed outcomes as complete remission, partial remission, no response, or nonadherence. Furthermore, it was challenging to separate patients with primary and secondary delusional infestation, because some studies combined analyses.

### Conclusion

Delusional infestation is a rare and challenging illness to treat. While antipsychotics are considered the mainstay treatment for primary delusional infestation, we found that SGAs, such as aripiprazole and risperidone, as well as SSRIs, led to higher rates of full remission than FGAs, such as haloperidol and pimozide. We recommend that clinicians take a detailed psychiatric history of patients with delusional infestation, as comorbid depression, anxiety, and trichotillomania may be better managed with SSRIs. Larger studies, such as randomized controlled trials, are required to better evaluate the effectiveness of SSRIs, FGAs, and SGAs for the treatment of delusional infestation.

## Abbreviations

FGA: first-generation antipsychotic
NIH: National Institutes of Health
SGA: second-generation antipsychotic
SSRI: selective serotonin reuptake inhibitor
